# Complete Genome Sequence of Porcine Sapelovirus Strain USA/IA33375/2015 Identified in the United States

**DOI:** 10.1128/genomeA.01055-16

**Published:** 2016-09-29

**Authors:** Qi Chen, Ying Zheng, Baoqing Guo, Jianqiang Zhang, Kyoung-Jin Yoon, Karen M. Harmon, Rodger G. Main, Ganwu Li

**Affiliations:** Department of Veterinary Diagnostic and Production Animal Medicine, College of Veterinary Medicine, Iowa State University, Ames, Iowa, USA

## Abstract

The complete genome of sapelovirus A, formerly known as porcine sapelovirus (PSV), from a diarrheic pig was sequenced for the first time in the United States (designated PSV USA/IA33375/2015). It shares 87.8% to 83.9% nucleotide identities with other reported PSV strains globally and is most closely related to Asia PSV strains.

## GENOME ANNOUNCEMENT

Porcine sapelovirus is a single-stranded, positive-sense, and nonenveloped RNA virus belonging to the genus *Sapelovirus* in the family *Picornaviridae* and consists of a single serotype, porcine sapelovirus 1 (PSV-1). Genetically the virus is closely related to members of the *Enterovirus* genus within the *Picornaviridae* family and was previously classified as porcine enterovirus A or 8. There are three species within the *Sapelovirus* genus: sapelovirus A (formerly known as porcine sapelovirus), sapelovirus B (formerly known as simian sapelovirus), and avian sapelovirus. Porcine sapelovirus has been detected in pigs with acute diarrhea, respiratory distress, reproductive failure, and/or polioencephalomyelitis (1), although PSV infection in swine can be asymptomatic (2).

In order to identify pathogens other than coronaviruses, such as transmissible gastroenteritis virus, porcine epidemic diarrhea virus (PEDV), and porcine deltacoronavirus in feces of pigs with acute diarrhea, metagenomics analysis was performed on 217 swine fecal samples from various geographic locations in the United States. Sequence of PSV was detected in 69 samples (31.8%). The complete genomic sequence of PSV (USA/IA33375/2015) identified in feces from a PEDV-positive pig with severe diarrhea was determined for the first time in the United States.

All clean reads from the fecal sample were classified using Kraken (3). The reads that were classified to sapelovirus A were then extracted and *de novo* assembled. The complete genome of PSV USA/IA33375/2015 is 7,565 nucleotides (nt) long and contains a 490-nt untranslated region (UTR) at the 5′ end, a 6,996-nt-long single open reading frame (491 to 7486) of the genome, and a 79-nt 3′ UTR. The putative polyprotein can be further cleaved into 12 proteins, including a leader protein (L), four structural proteins (VP4, VP3, VP2, and VP1), and seven nonstructural proteins (2A, 2B, 2C, 3A, 3B, 3C, and 3D). The genome of PSV USA/IA33375/2015 shares 87.8% to 83.9% nucleotide identities with that of seven other global PSV strains (one from the United Kingdom, three from China, and three from South Korea) identified from 2002 to 2014 whose whole-genome sequences are currently available in GenBank (accession numbers JX286666, HQ875059, KF539414, KJ821021, KJ821020, KJ821019, and AF406813). Phylogenetically, the PSV USA/IA33375/2015 strain is clustered together with the Korean (GenBank accession numbers KJ821021, KJ821020, and KJ821019) and Chinese (GenBank accession numbers JX286666, HQ875059, and KF539414) PSV-1 strains, which are separated from the United Kingdom PSV-1 V13 strain (GenBank accession number AF406813).

Porcine sapelovirus has been reported in Europe, Asia, and South America (4–7); however, to our best knowledge, the virus from U.S. swine has not been fully characterized molecularly. Although the clinical significance of PSV remains to be determined, our first complete genome sequence of U.S. PSV will facilitate future studies in diagnostics and molecular epidemiology of PSV in U.S. swine.

### Accession number(s).

The complete genome sequence of PSV USA/IA33375/2015 has been deposited in GenBank under accession number KX574284.
